# Self-Adjuvanting Glycopeptide Conjugate Vaccine against Disseminated Candidiasis

**DOI:** 10.1371/journal.pone.0035106

**Published:** 2012-04-26

**Authors:** Hong Xin, Jonathan Cartmell, Justin J. Bailey, Sebastian Dziadek, David R. Bundle, Jim E. Cutler

**Affiliations:** 1 Department of Pediatrics, Louisiana State University Health Sciences Center and Research Institute for Children, Children's Hospital, New Orleans, Louisiana, United States of America; 2 Alberta Ingenuity Centre for Carbohydrate Science, Department of Chemistry, University of Alberta, Edmonton, Alberta, Canada; University of Aberdeen, United Kingdom

## Abstract

Our research on pathogenesis of disseminated candidiasis led to the discovery that antibodies specific for *Candida albicans* cell surface β-1, 2–mannotriose [β-(Man)_3_] protect mice. A 14 mer peptide Fba, which derived from the *N*-terminal portion of the *C. albicans* cytosolic/cell surface protein fructose-bisphosphate aldolase, was used as the glycan carrier and resulted in a novel synthetic glycopeptide vaccine β-(Man)_3_-Fba. By a dendritic cell-based immunization approach, this conjugate induced protective antibody responses against both the glycan and peptide parts of the vaccine. In this report, we modified the β-(Man)_3_-Fba conjugate by coupling it to tetanus toxoid (TT) in order to improve immunogenicity and allow for use of an adjuvant suitable for human use. By new immunization procedures entirely compatible with human use, the modified β-(Man)_3_-Fba-TT was administered either alone or as a mixture made with alum or monophosphoryl lipid A (MPL) adjuvants and given to mice by a subcutaneous (s.c.) route. Mice vaccinated with or, surprisingly, without adjuvant responded well by making robust antibody responses. The immunized groups showed a high degree of protection against a lethal challenge with *C. albicans* as evidenced by increased survival times and reduced kidney fungal burden as compared to control groups that received only adjuvant or DPBS buffer prior to challenge. To confirm that induced antibodies were protective, sera from mice immunized against the β-(Man)_3_-Fba-TT conjugate transferred protection against disseminated candidiasis to naïve mice, whereas *C. albicans*-absorbed immune sera did not. Similar antibody responses and protection induced by the β-(Man)_3_-Fba-TT vaccine was observed in inbred BALB/c and outbred Swiss Webster mice. We conclude that addition of TT to the glycopeptide conjugate results in a self-adjuvanting vaccine that promotes robust antibody responses without the need for additional adjuvant, which is novel and represents a major step forward in vaccine design against disseminated candidiasis.

## Introduction

Hematogenously disseminated candidiasis in humans has become the third or fourth leading cause of hospital-acquired blood stream infections and despite antifungal therapy at least 40% of affected individuals will die of this disease [1], [2]. It's estimated that 60–70,000 cases of disseminated candidiasis occur per year in the US alone, and associated health care costs are $2–4 billion/year [3]. The limited number and toxicity of antifungal agents, and, most importantly, the poor outcome of almost half of the number of candidemia patients treated with appropriate antifungal therapy, militates in favor of disease prevention, possibly through active and passive immunization strategies [4]–[6]. Strong evidence has accumulated in the last decade that antibodies specific for certain cell surface epitopes of fungi may be beneficial for the fungal-infected host [7]–[13]. In addition, if a vaccine maintains a long-lived protective antibody titer, we argue that this form of disease prevention could be induced and protect individuals who will enter into a possible transient immunocompromised state, such as those patients who will have elective abdominal or other surgical procedures that will place them at risk of developing candidiasis.

Depending on the clinical setting there is a wide spectrum of *Candida* species that may cause disseminated candidiasis, but *C. albicans* continues to be prevalent overall and this species is the most virulent in experimental animals [14], [15]. Antibodies have long been considered irrelevant in host defense against invasive candidiasis, but over the last two decades a number of antibodies or their engineered derivatives directed against *C. albicans* cell-wall polysaccharides and glycopeptides, as well as against some protein or peptide epitopes, have been shown to confer protection [6], [12], [16], [17]. We previously demonstrated that complement-fixing antibodies that recognize *C. albicans* cell surface β-1,2-linked mannotriose [β-(Man)_3_] protect mice against candidiasis [18], [19]. Our finding that a *C. albicans* cell surface peptide Fba, derived from the N-terminal portion of *C. albicans* cell wall protein fructose-bisphosphate aldolase, may serve as an immunologic carrier for the glycan has resulted in a novel fully synthetic glycopeptide vaccine [7]. Following immunizations of mice, protection was afforded by antibodies specific for the β-(Man)_3_ and the Fba epitopes that comprised the vaccine [7]. The antibody dependency of protection was evident by protection transferred to naïve mice by immune serum, but not by serum pre-absorbed with *C. albicans*. These results enabled elucidation of an efficacious vaccine, but the immunization protocol utilized dendritic cells and complete Freund adjuvant (DC/CFA), which are cost prohibitive and incompatible for human use, respectively. In this report, we have investigated alternative vaccine presentation strategies to test our hypothesis that an approved human adjuvant can be substituted for the DC/CFA approach provided that the vaccine conjugate can be appropriately modified to improve immunogenicity.

Prior to vaccine modification, we expanded our observation on efficacy of the β-(Man)_3_-Fba vaccine in additional mouse strains and against challenge with an additional *C. albicans* strain, and tested whether the vaccine could be administered with alum or monnophosphoryl lipid A (MPL) adjuvants in place of DC/CFA. In subsequent experiments, the vaccine modification was the covalent coupling of tetanus toxoid (TT) to the β-(Man)_3_-Fba. The β-(Man)_3_-Fba-TT conjugate was administered alone or as a mixture made with alum or MPL. The best protection results occurred in animals immunized against the β-(Man)_3_-Fba-TT conjugate vaccine with, or, surprisingly, without additional adjuvant. This self-adjuvanting β- (Man)_3_-Fba-TT conjugate vaccine, administered without any additional adjuvant, induced robust antibody responses and antibody-mediated protection in mice.

## Results

### Protective efficacy of β-(Man)_3_-Fba conjugate vaccine in a different mouse strain and against an additional *C. albicans* strain

As we described, the β-(Man)_3_-Fba conjugate vaccine induced strong antibody responses and protective immunity in BALB/c mice [7] that express the H-2^d^ MHC haplotype and have a Th-2 immunologic bias [20], [21]. C57BL/6 mice express an H-2^b^ MHC haplotype, are more prone to Th1 responses and supposedly more resistant to disseminated candidiasis than are BALB/c mice [21]–[23]. We derived dendritic cells in vitro as described before [7] and used the same immunization DC/CFA-strategy on the C57BL/6 mice as was used in our work on BALB/c mice [7], which included a priming dose followed by two boosters; the last booster consisted of the vaccine emulsified in CFA. C57BL/6 mice responded to the vaccine by making specific antibody against each of the two vaccine epitopes, i.e., the β-(Man)_3_ and the Fba peptide (data not shown). Following the first booster, an isotype switch from IgM to IgG occurred in response to each epitope. The immunized C57BL/6 mice showed 80% survival throughout the 120 days post challenge and survived significantly longer (*p*<0.001) as compared to the control groups of mice given DPBS buffer, DC or DC+CFA (Figure S1A). The survival data were consistent with the trend of colony forming units (CFU) in kidney homogenates. That is, immunized C57BL/6 mice had greatly reduced or non-detectable kidney CFU as compared to controls that were sacrificed when they became moribund following i.v. challenge with the fungus (Figure S1B). Indeed, the protection in C57BL/6 mice was similar to that which we observed for BALB/c mice [7].

To answer whether antibody responses were responsible for the protection, antisera were collected from separate groups of immunized mice and transferred i.p. to naïve mice 4 h before i.v. challenge with a lethal dose of *C. albicans* strain 3153A. Control groups were given either immune sera pre-absorbed with live *C. albicans* yeast cells or DPBS buffer prior to the challenge. The immune serum donors, which were immunized with β-(Man)_3_-Fba by the DC/CFA method, were used as positive controls for protection. After challenge, immunized mice and mice treated with the antiserum had prolonged survival times as compared to the two control groups (*p*<0.05) (Figure S1C), and as expected, mice that received the antiserum had significantly reduced fungal counts in their kidneys (*p*<0.05) (Figure S1D). These data provide strong evidence that antibodies are responsible, at least in part, for the vaccine-induced protection against a lethal challenge with the fungus in C57BL/6 mice.

*C. albicans* strain 3153A was used in our previous studies [7], [17], [19]. To test if DC/CFA vaccination with the β-(Man)_3_-Fba protects C57BL/6 mice challenged with another *C. albicans* strain, we challenged immunized mice with *C. albicans* strain SC5314, a clinical isolate commonly used in research. As a positive control, a group of immunized mice was challenged with strain 3153A. Similar protection patterns were observed in both groups of mice regardless of the challenge strain (Figure S1E). In addition to prolonged survival times, immunized groups had reduced or non-detectable CFUs in their kidneys as compared to non-immune mice (data not shown). These results are similar to those we observed from BALB/c mice challenged with the 3153A strain [7], [19]. The above experiments are important as they show that vaccine-induced antibody protection is not animal or fungal-strain dependent.

### Immunization with β-(Man)_3_-Fba combined with alum or MPL induced modest antibody responses and slight protection

**Figure 1:**
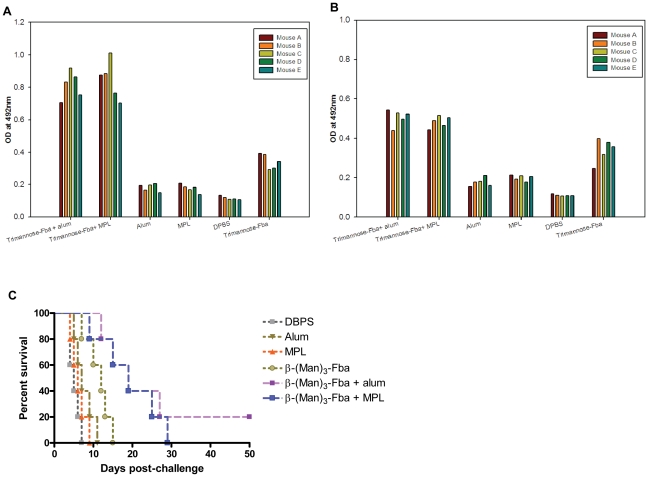
β-(Man)_3_-Fba administered along with either alum or MPL adjuvants induced modest antibody responses and slight protection against disseminated candidiasis. The β-(Man)_3_-Fba conjugate was administered as a mixture with either alum or MPL adjuvants in BALB/c mice. Serum samples were collected 14 days after immunization, diluted 1∶100 and tested by ELISA on plates coated with synthetic β-(Man)_3_ or Fba-MAP. After the first booster immunization, immune sera from vaccinated mice showed modest antibody responses to Fba peptide (A) and relatively weak antibody responses to β-(Man)_3_ epitope (B) (C) The survival was also slightly extended in mice that received β-(Man)_3_-Fba in MPL and slight protection was observed when alum was used as the adjuvant as compared to DPBS or adjuvant unimmunized controls.

Although the DC/CFA -based immunization approach was successful in mice for protection against disseminated candidiasis, the use of DC and complete Freund adjuvant are inappropriate for human use. To test new adjuvants suitable for human use, the β-(Man)_3_-Fba conjugate was administered as a mixture with either alum or MPL adjuvants. After the first booster, immune sera from vaccinated mice showed modest antibody responses (OD values of a 1/100 serum dilution: 0.7–0.9) to the Fba peptide (Figure 1A), and relatively weak antibody responses (OD values of a 1/100 serum dilution: 0.45–0.55) to the β-(Man)_3_ epitope (Figure 1B). Following the second booster immunization, an isotype switch from IgM to IgG of either β-(Man)_3_ or Fba specific antibodies was low to negligible in immune sera (data not shown, results summarized in table 1), which suggested that an immune memory response had not occurred. In addition, the β-(Man)_3_-Fba vaccinated groups had insignificantly longer survival times as compared to the two non-immunized control groups after challenge with a lethal dose of *C. albicans* cells (*p* = 0.77) (Figure 1C).

**Table 1:** Antibody isotype distribution of responses to Fba and β-(Man)_3_.

Sera induced by vaccines	anti β-(Man)_3_	anti Fba-peptide
β-(Man)3-Fba-TT with MPL	IgM;IgG1;IgG2a;IgG2b	IgM;IgG1
β-(Man)_3_-Fba-TT with alum	IgM;IgG1;IgG2a	IgM;IgG1;IgG2a
β-(Man)_3_-Fba-TT	IgM;IgG1;IgG2a	IgM;IgG1;IgG2a
β-(Man)_3_-Fba	IgM	IgM
β-(Man)_3_-Fba with alum	IgM	IgM
β-(Man)_3_-Fba with MPL	IgM	IgM
β-(Man)_3_-Fba+DC+CFA	IgM;IgG1	IgM;IgG1

**Figure 2:**
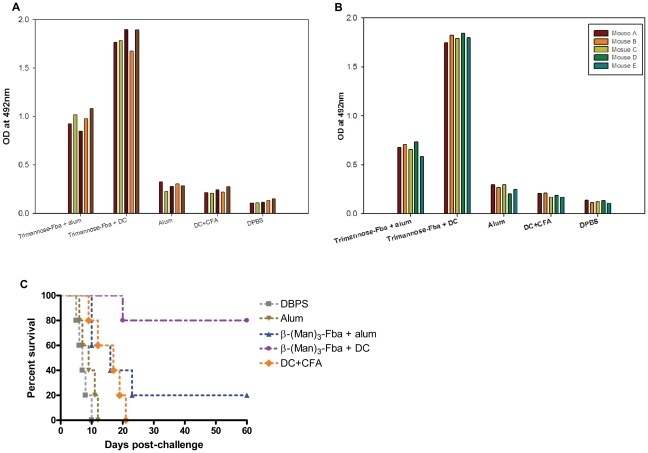
Comparison of DC/CFA and alum as adjuvants for induction of immune resopnses to the β-(Man)_3_-Fba conjugate vaccine. Serum samples were collected 14 days after immunization, diluted 1∶100 and tested by ELISA on plates coated with either synthetic Fba-MAP or β-(Man)_3_. Immune sera from mice immunized with the β-(Man)_3_-Fba DC/CFA showed greater antibody titers to both the Fba peptide (A) and the β-(Man)_3_ epitopes (B) than sera from groups that received β-(Man)_3_-Fba in alum. (C) A high degree of protection was induced by the β-(Man)_3_-Fba pulsed DCs, and slight protection was observed when alum was used as the adjuvant as compared to DPBS, DC+CFA or alum adjuvant unimmunized controls.

In an attempt to increase the antibody and protective responses, the dose of β-(Man)_3_-Fba conjugate was increased from 2.5 µg to 10 µg in the β-(Man)_3_-Fba+alum formulation. Nonetheless, the levels of anti-Fba peptide (Figure 2A) and anti-β-(Man)_3_ (Figure 2B) were markedly less than antibody levels (OD values of a 1/100 serum dilution: 1.7–1.9) in sera from control animals immunized with the β-(Man)_3_-Fba+DC/CFA. Likewise, when titers were assessed by end-point dilution, the immune sera from the positive control group showed significantly greater antibody responses for both epitopes (Table 2 & 3). Interestingly, even though the antibody titers against both epitopes in response to the 10 microgram dosage was greater than the response to the 2.5 µg dose, disease protection was not observed to the extent of protection induced by the DC/CFA immunization approach (Figure 2C). In summary, the greatest antibody responses occurred in mice that received the β-(Man)_3_-Fba+DC/CFA, the animals of which also showed evidence of an IgM-IgG shift (Table 2 & 3) and the highest degree of protection [7].

**Table 2:** ELISA titers against microtiter wells coated with synthetic β-(Man)_3_ epitope.

Sera induced by vaccines	anti β-(Man)_3_ ELISA titers* (*n = 5 mice per group)				
β-(Man)_3_-Fba-TT with MPL	I2,800	25,600	25,600	25,600	25,600
β-(Man)_3_-Fba-TT with alum	I2,800	12,800	25,600	25,600	25,600
β-(Man)_3_-Fba-TT	I2,800	25,600	25,600	12,800	25,600
β-(Man)_3_-Fba with alum	I,600	400	400	400	800
β-(Man)_3_-Fba with MPL	I,600	800	800	400	400
β-(Man)_3_-Fba	400	400	400	400	400
β-(Man)_3_-Fba+DC+CFA	51,200	25,600	25,600	25,600	51,200

**Table 3:** ELISA titers against microtiter wells coated with synthetic Fba peptide.

Sera from vaccine groups	anti- Fba peptide ELISA titers* (*n = 5 mice per group)				
β-(Man)_3_-Fba-TT with alum	51,200	25,600	51,200	51,200	25,600
β-(Man)_3_-Fba-TT with MPL	51,200	25,600	25,600	25,600	25,600
β-(Man)_3_-Fba-TT	51,200	25,600	25,600	51,200	25,600
Fba-TT with alum	400	400	200	N/A	N/A
Fba-TT with MPL	400	400	200	N/A	N/A
β-(Man)_3_-Fba with alum	800	800	1600	800	800
β-(Man)_3_-Fba with MPL	800	800	800	400	800
β-(Man)_3_-Fba	400	400	400	400	400
β-(Man)_3_-Fba+DC+CFA	51,200	102,400	51,200	51,200	102,400

### Addition of tetanus toxoid (TT) to the vaccine, β-(Man)_3_-Fba-TT, markedly enhanced antibody responses to both epitopes in the presence of alum or MPL

**Figure 3:**
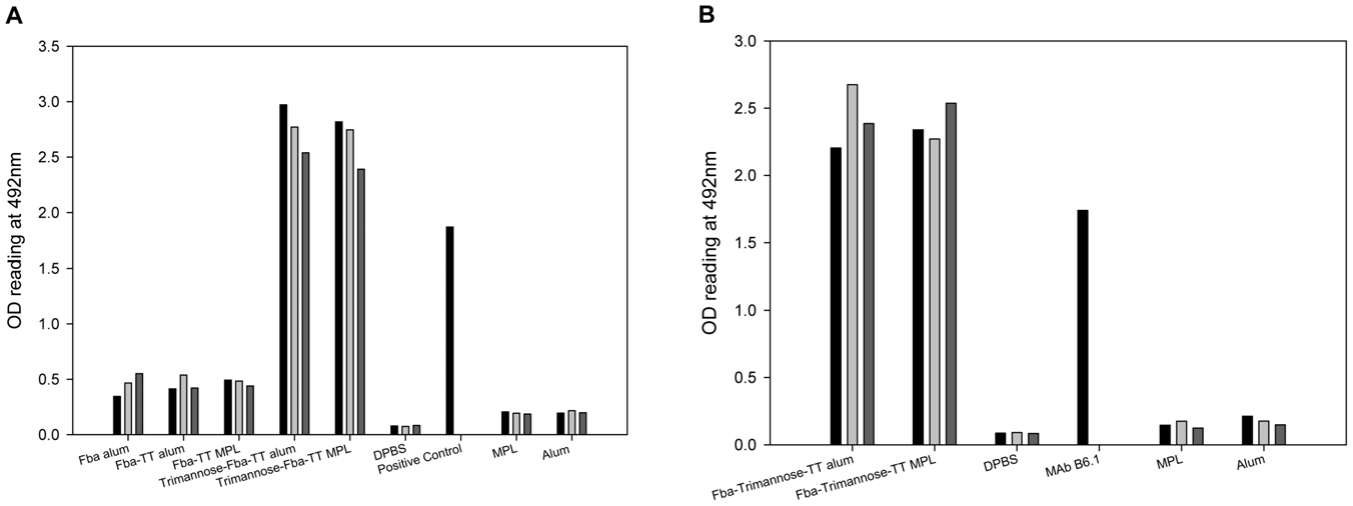
Vaccination with β-(Man)_3_-Fba-TT in either alum or MPL markedly increased both β-(Man)_3_ and Fba peptide-specific antibody titers in sensitized mice as compared to controls. Serum samples were collected 14 days after immunization, diluted 1∶100 and tested by ELISA on plates coated with cell wall mannan or peptide. MAbs B6.1 and E2-9 that are specific for β-(Man)_3_ and Fba, respectively, were used as positive controls. Mice immunized with β-(Man)_3_-Fba-TT prepared in either alum or MPL induced robust antibody responses against both the Fba peptide (A) and the β-(Man)_3_ (B) epitopes. However, mice that received either Fba or Fba-TT in either adjuvant produced weak anti-Fba responses.

In an attempt to improve immunogenicity of the glycopeptide vaccine in the presence of adjuvant suitable for human use, we modified the β-(Man)_3_-Fba conjugate by coupling it to tetanus toxoid designated as β-(Man)_3_-Fba-TT. In a preliminary experiment we also tested the Fba peptide as a Fba-TT conjugate. Both conjugates were administered as mixtures with alum or MPL. Negative control groups included adjuvant only and DPBS buffer only. After the first booster, mice immunized with β-(Man)_3_-Fba-TT prepared in either alum or MPL produced robust antibody responses against both the Fba peptide (Figure 3A) and the β-(Man)_3_ epitopes (Figure 3B), titers of which were 100 fold greater than that of sera from groups that received Fba-TT (*p*<0.001) (Table 2 & 3), the latter of which responded about the same as animals that received Fba in alum without TT (Figure 3A). After the first booster, IgM and IgG antibodies against both epitopes were detected in the sera of mice immunized with β-(Man)_3_-Fba-TT with added alum or MPL adjuvants (Table 1), whereas very low levels of anti-Fba IgM and IgG antibodies were detected in the sera of mice that received Fba or Fba-TT in adjuvant. No antibody against the epitopes was detectable in any of the negative (i.e., adjuvant or DPBS mice) control sera (data not shown).

### The β-(Man)_3_-Fba-TT conjugate vaccine induced high antibody responses and protection even in the absence of adjuvant

**Figure 4:**
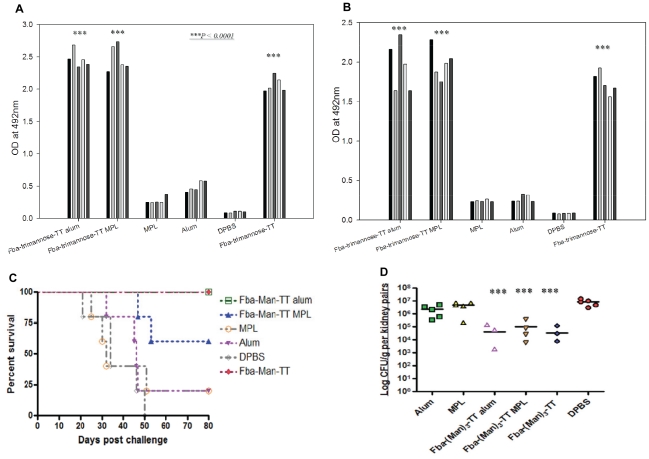
β-Man)_3_-Fba-TT conjugate with or without adjuvant markedly induced high antibody titers and protection against disseminated candidiasis in immunized mice as compared to controls. Mice immunized with β-(Man)_3_-Fba-TT prepared in either alum or MPL, or without adjuvant developed robust antibody responses against both the Fba peptide (A) and the β-(Man)_3_ epitope (B). (C) Protective immunity was induced by (β-Man)_3_-Fba-TT when either alum or MPL was used as the adjuvant. Protection was nearly as great even when adjuvant was omitted as compared to DPBS or adjuvant only controls (*P*<0.01). (D) Immunized mice had reduced or non-detectable CFUs per kidney pairs compared to control groups (*P*<0.001).

To determine whether the immunogenicity of β-(Man)_3_-Fba-TT vaccine was dependent on additional adjuvant, β-(Man)_3_-Fba-TT was administered alone and the response of these mice was compared to those that received the vaccine as a mixture made with alum or MPL adjuvants. Mice that received the vaccine prepared in either adjuvant responded as expected by making robust antibody responses. Surprisingly, mice that received the β-(Man)_3_-Fba-TT without adjuvant responded only slightly, but not significantly, less than those that received the vaccine plus adjuvant (Figure 4A+4B). Importantly, all three groups of mice, vaccinated with β-(Man)_3_-Fba-TT conjugate vaccine with or without additional adjuvant, showed a high degree of protection against a lethal challenge with *C. albicans* (Figure 4C). The induced protective immunity was evidenced by significantly prolonged survival times (*p*<0.005)and reduced kidney fungal burden (*p*<0.001) as compared to control groups that received only adjuvants or DPBS buffer prior to challenge (Figure 4D). These results showed the self-adjuvanticity power of the β-(Man)_3_-Fba-TT vaccine.

### Anti-β-(Man)_3_-Fba-TT immune sera induced by non-DC/CFA-based immunization approaches provided passive protection

**Figure 5:**
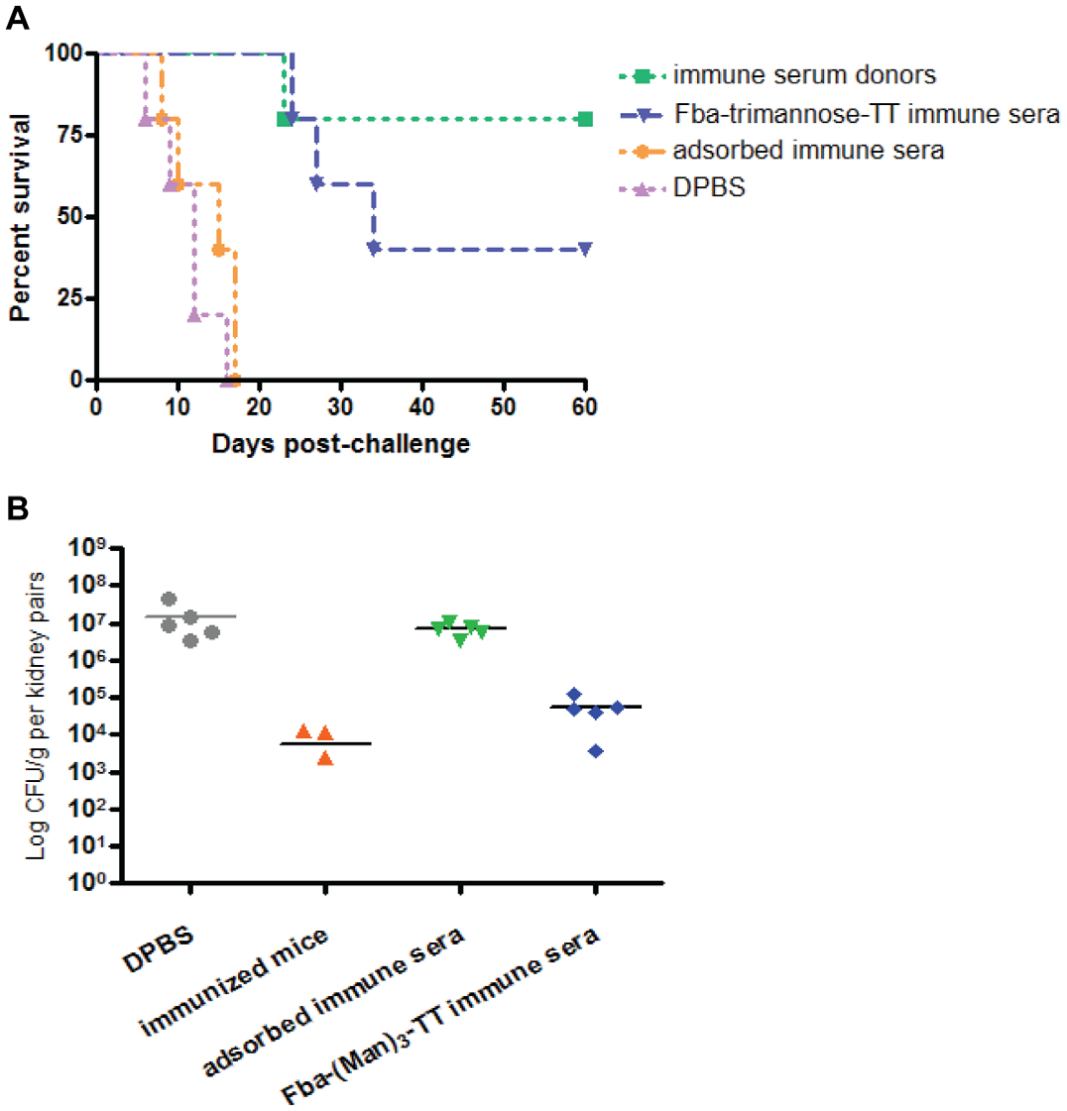
Passive transfer experiment was performed to confirm that antibody is responsible for the protection. (A) As immunized mice, immune sera recipients had a prolonged survival time (*P*<0.01), confirming that induced antibodies were protective. (B) The serum from (β-Man)_3_-Fba-TT immunized animals was capable of reducing the fungal load in the mouse kidneys compared with the infectious burden in mice were given DPBS or pre-absorbed sera (*P*<0.001).

In previous work we showed by passive transfer experiments that antibodies induced by the DC/CFA-based immunization approach are responsible for protection against disseminated candidiasis. To confirm that vaccine-induced antibodies are protective regardless of the use of dendritic cells, immune sera were collected and pooled from β-(Man)_3_-Fba-TT (with or without alum or MPL adjuvants) immunized mice and transferred i.p. to naïve mice 4 h before i.v. challenge with a lethal dose of *C. albicans*. Control groups were given either immune serum pre-absorbed with live *C. albicans* yeast cells or DPBS buffer prior to the challenge. We tested for antibodies against the β-(Man)_3_ and Fba eptiopes before and after absorption with yeast cells. Immune serum donors, which were immunized with β-(Man)_3_-Fba-TT conjugate vaccine, were used as a positive control for protection. After challenge, immunized positive control mice and mice treated with the antiserum had prolonged survival times as compared to the two negative control groups (*p*<0.01) (Figure 5A), confirming that induced antibodies were protective and that their induction was not dependent on the use of dendritic cells or CFA during the immunizations. Consistently, mice that received the antiserum had significantly fewer fungal counts in their kidneys compared with the infectious burden in mice that were given DPBS or pre-absorbed serum prior to challenge (*p*<0.001) (Figure 5B).

### Immunization induced an isotype switch from IgM to IgG for antibodies specific for either fungal epitope in the vaccine conjugate

We compared antibody isotype responses to both the glycan and peptide epitopes induced by the β-(Man)_3_-Fba conjugate when the DC/CFA-based immunization approach was employed to that produced by the β-(Man)_3_-Fba-TT modified conjugate administered with alum or MPL or when given alone (Table 1). β-(Man)_3_-Fba+DC/CFA and β-(Man)_3_-Fba-TT immunized mice produced antibodies to both the β-(Man)_3_ epitope and Fba peptide, and the isotype analysis revealed an abundance of IgM and IgG subclasses in the immune sera against both epitopes, which is consistent with the induction of a T cell-dependent memory immune response.

The isotype distribution of antibodies specific for the fungal epitopes differed depending on the adjuvant system (Table 1). Whereas IgM and IgG1 responses to β-(Man)_3_ were induced regardless of the presence of adjuvant, an IgG2a response to the glycan epitope was induced by β-(Man)_3_-Fba-TT with or without the use of alum or MPL, but IgG1 was the only subclass detectable in mice immunized by the DC/CFA approach. Only mice immunized with the β-(Man)_3_-Fba-TT mixed with MPL produced an IgG2b response to the glycan epitope. Antibody IgM and IgG1 isotype responses to the Fba peptide were similar for mice immunized with the β-(Man)_3_-Fba regardless of the adjuvant system, however, IgG2a specific for the peptide epitope was induced only by β-(Man)_3_-Fba-TT with alum or when no adjuvant was used. No IgG2a isotype was detected against the peptide when MPL was the test adjuvant. The level of protection observed against disseminated candidiasis was similar in mice immunized with the glycan-peptide conjugate in the DC/CFA approach, or in mice immunized with the glycan-peptide-TT with or without alum or MPL, which indicated that the protective antibodies are likely to be primarily of the IgM and IgG1 isotypes.

### The glycopeptide-TT conjugate is immunogenic and protective against disseminated candidiasis in outbred mice

**Figure 6:**
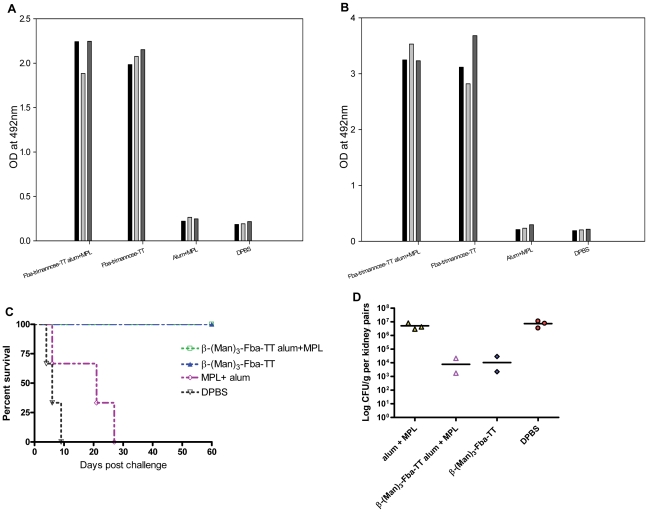
The (β-Man)_3_-TT conjugate vaccine is immunogenic and protective against disseminated candidiasis in outbred mice. Outbred CFWSwiss Webster (01S60) mice were immunized with the β-(Man)_3_-Fba-TT conjugate alone or mixed with adjuvants alum and MPL; control mice were immunized with adjuvants (alum+MPL) only or DPBS buffer. Mice immunized with β-(Man)_3_-Fba-TT in either alum+MPL, or without adjuvant induced robust antibody responses against both the β-(Man)_3_ epitope (A) and the Fba peptide (B). (C) Protective immunity was induced by (β-Man)_3_-Fba-TT with or without adjuvant as noted by their prolonged survival time as compared to control mice that received DPBS or adjuvants alone (*P*<0.01). (D) Immunized mice had reduced or non-detectable CFUs per kidney pairs compared to control groups.

The efficacy of the β-(Man)_3_-Fba-TT conjugate vaccine against disseminated candidiasis was also demonstrated in outbred mice. Since a combination of MPL and alum may enhance the vaccine response by rapidly triggering a local cytokine response leading to an optimal activation of APCs [24], the tested Swiss Webster mice were immunized with the β-(Man)_3_-Fba-TT conjugate alone or as a mixture with both alum and MPL. Negative control mice were immunized with the adjuvant combination or DPBS only. The β-(Man)_3_-Fba-TT, with or without adjuvant, induced robust and consistent antibody responses against both the β-(Man)_3_ (Figure 6A) and the Fba epitopes (Figure 6B) in immunized outbred mice. Fourteen days following the last booster, immunized mice were infected via the tail vein with a lethal dose of *C. albicans* 3153A, as we have described previously. Similar to our previous findings in BALB/c mice, the outbred mice vaccinated with β-(Man)_3_ –Fba-TT conjugate vaccine, when administered alone or with alum and MPL, markedly improved the survival of infected mice (Figure 6C). Consistently, the immunized mice had significantly lower live fungal cells in their kidneys as compared to negative controls (*p*<0.001) (Figure 6D).

### Antibodies in immune sera bind yeast and hyphal forms of *C. albicans*

**Figure 7:**
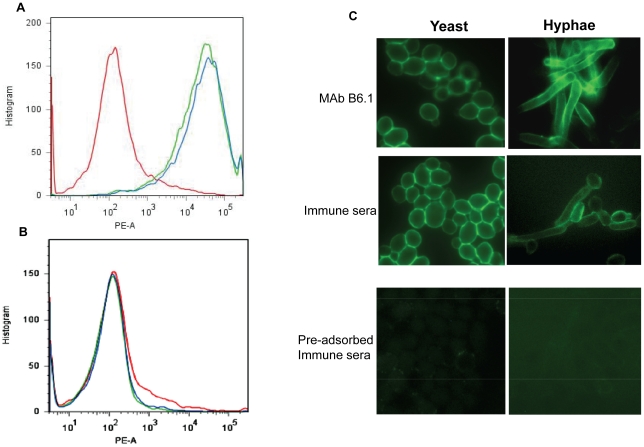
Immune serum from (β-Man)_3_-Fba-TT vaccinated mice detected the presence of the vaccine epitopes on the surface of *C. albicans*. (A) Antibodies in immune sera binding to the both epitopes expressed on the *C. albicans* cell surface were confirmed by flow cytometry. The reactivity of immune serum (green) with live *C. albicans* cells to that of MAb B6.1 (blue line), which is specific for the *C. albicans* cell surface epitope (β-Man)_3_,. Control serum (red line) was non-immune serum from mice that received adjuvant only. (B) Pre-absorbed MAb B6.1 (blue line) and pre-absorbed immune sera (green line) were not reactive with fungal cell surface. (C) Confocal microscopic analyses confirmed that antibodies in immune serum detect the vaccine epitopes on the surface of yeast forms, but are also reactive with the surface of hyphal forms of *C. albicans*. The epitope display was similar to that due to fungal reactivity with MAb B6.1, which is specific for β-(Man)_3_ and was used as a positive immunofluorescence control. As an additional negative control, immune serum pre-absorbed with *C. albicans* 3153A yeast cells did not react with either yeast or hyphal forms of *C. albicans*.

Immune serum from animals immunized with the β-(Man)_3_-Fba-TT conjugate contained antibodies specifically reactive with the cell surface of yeast forms as demonstrated by flow cytometric analyses. A fluorescence shift similar in magnitude to the control antibody, MAb B6.1, was observed upon testing of the immune serum (Figure 7A). This reactivity of immune serum was removed by pre-absorption with *C. albicans* yeast forms (Figure 7B). The binding pattern was similar with immune sera collected from mice vaccinated with β-(Man)_3_-Fba-TT alone, or when mixed with either alum or MPL (data not shown).

Microscopic observations after immunofluorescence staining with anti-β-(Man)_3_-Fba-TT conjugate immune serum showed reactivity with both yeast and filamentous forms of *C. albicans* strain 3153A (Figure 7C), which was expected since we previously showed that the vaccine glycan and peptide epitopes are surface expressed [7], [17], [18]. The microscopic analysis confirmed the flow cytometry results and extended the observations to include hyphal forms of the fungus. The specific antibody reactivity was again confirmed by the absence of fluorescence by immune serum pre-absorbed with yeast forms of the fungus (Figure 7C). As with the flow cytometry analyses, the positive reaction of immune serum compared favorably with reactivity of MAb B6.1, which is specific for the glycan epitope [25], [26]. Moreover, essentially the same pattern of *C. albicans* fluorescence was observed with immune serum from mice vaccinated with β-(Man)_3_-Fba-TT alone, or when mixed with alum/MPL. No reactivity with the fungus was observed upon testing of serum from mice given adjuvant only or pooled serum from untreated normal mice. In addition to these findings, serum from mice immunized with the β-(Man)_3_-Fba-TT conjugate reacted similarly with another *C. albicans* isolate (strain SC5314) (data not shown).

## Discussion

The discovery of numerous antigens on the fungal cell wall that elicit protective antibody responses raises the possibility of obtaining combined or even synergistic efficacious effects of vaccines designed with multiple antigens, and/or passive therapies that combine antibodies with different specificities [27], [28]. The novel fully synthetic β-(Man)_3_-Fba glycopeptide vaccine, which is based on two *C. albicans* cell surface epitopes, induces impressive protection against disseminated candidiasis in BALB/c mice [7]. Furthermore, in previous work we provided strong evidence that antibodies specific for the glycan and peptide epitopes contribute to the protection [17], [29], [30].

In the present study we extended our observations to include C57BL/6 mice, which are more prone to Th1 responses and more resistant to disseminated candidiasis as compared to the BALB/c mice. By the same DC/CFA-based immunization protocols that favored production of protective antibody, the β-(Man)_3_-Fba conjugate induced a similar level of protection in C57BL/6 mice as we found for BALB/c animals. Furthermore, protection was observed regardless of the challenge strain of *C. albicans*. As with the BALB/c mice, passive transfer of antibodies against the two fungal epitopes to C57BL/6 naïve mice protected these animals against disseminated candidiasis.

For the present and previous studies, the antigen-pulsed DC/CFA immunization approach proved to be a powerful way of overcoming the relatively weak immune response of the mouse to the defined small glycan and peptide antigens that comprised the vaccine. This immunization approach enabled us to arrive at the defined vaccine composition [β-(Man)_3_-Fba] that provides a high degree of protection against experimental hematogenously disseminated candidiasis. The critical limitation of the immunization approach is the use of DC and CFA, the former of which would severely limit widespread vaccine use in humans and the latter of which is unacceptable for human use. For those reasons we tested the β-(Man)_3_-Fba along with alum and MPL as adjuvants in place of DC and CFA. However, relatively weak anti-β-(Man)_3_ and moderate anti-Fba responses were induced. Perhaps more importantly, the new immunization approaches failed to induce an IgM to IgG isotype shift, suggesting the lack of a memory cell response. In addition, only the mice immunized with a combination of the β-(Man)_3_-Fba conjugate and alum showed evidence of protection, and even that group of animals was not protected nearly as well as animals immunized by the DC/CFA approach. The results did not improve by increasing the dose of the β-(Man)_3_-Fba conjugate from 2.5 µg to 10 µg, which led us to investigate modifications of the vaccine itself.

In an attempt to increase the immunogenicity of the β-(Man)_3_-Fba conjugate when using an acceptable immunization approach for human use, we investigated the effects of coupling the conjugate to tetanus toxoid (TT). The new glycopeptide vaccine conjugate, β-(Man)_3_-Fba-TT, proved to be highly immunogenic as it induced robust antibody responses when administered with either alum or MPL as adjuvants. Moreover, prior to the second booster dose, an isotype switch occurred from IgM to IgG antibodies against for both the β-(Man)_3_ and Fba peptide epitopes. This result indicated a possible memory cell response and, perhaps, a vaccine that induces long-term immunity. Most importantly, the (β-Man)_3_-Fba-TT conjugate administered with either alum or MPL induced protection against disseminated candidiasis on a par with the high level of protection observed with the original DC/CFA immunization approach [7].

**Figure 8:**
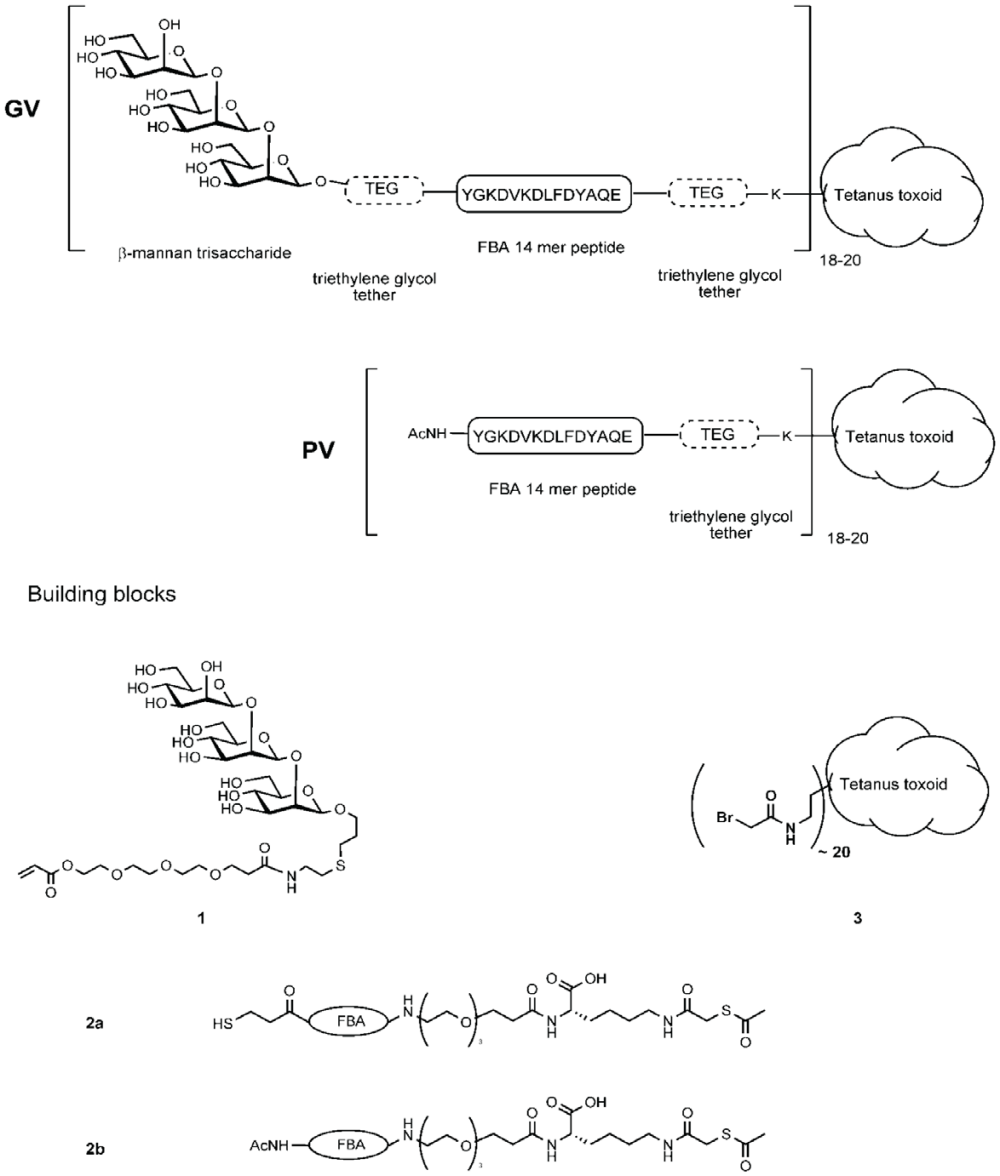
Scheme for synthesis of the conjugate vaccine. The glycoconjugate vaccine (**GV**) and the peptide vaccine lacking the mannotriose component (**PV**) were synthesized from the advanced building blocks **1**–**3**. The β-1,2 mannotriose derivatized with a triethylene glycol spacer **1**; the T-cell tetradecameric peptide (Fba) was assembled on a peptide synthesizer and a triethylene glycol tether was introduced at the C terminal end followed by a single lysine residue which was derivatized on its side chain by a thioacetic acid residue, which gave the building blocks **2a** or **2b**. Bromoacetate groups were introduced on approximately 20 of the lysine residues present in tetanus toxoid to give **3**. **GV** was assembled by reacting **1** with **2a** and then conjugating this product with **3**. **PV** was prepared by conjugating 2b with 3. A detailed account of this synthesis will be reported elsewhere (Cartmell *et al.* Carbohydr. Res submitted).

In previous work involving the DC/CFA immunization approach, we found that the Fba peptide itself was immunogenic, inducing not only a robust antibody response, but the response was protective as well against disseminated candidiasis [8], [17]. We were surprised, therefore, to find that the Fba-TT conjugate in adjuvant did not perform well in mice (Figure 3A & Table 3). We do not yet know the reason for this, but possibilities to consider include immune interference caused by the acetylated modification at the N-terminus (Figure 8) and, possibly, modifications may be required of the tether between the small Fba moiety and the TT.

The large differences in vaccine efficacy between glycopeptide conjugates β-(Man)_3_-Fba and β-(Man)_3_-Fba-TT when the same adjuvant system was used led us to consider whether the tetanus toxoid component was sufficient for inducing a protective immune response. To test this hypothesis, β-(Man)_3_-Fba-TT administered alone was compared to administration of the conjugate as either a mixture made with alum or MPL. Mice that received the β-(Man)_3_-Fba-TT conjugate prepared in either adjuvant responded as expected by making robust antibody responses. Surprisingly, mice that received the β-(Man)_3_-Fba-TT without any adjuvant also responded well. All three groups of vaccinated mice showed a high level of protection against a lethal challenge with *C. albicans* as evidenced by significantly increased survival times and reduced or non-detectable kidney fungal burden at the time of sacrifice as compared to control groups that received only adjuvants or DPBS buffer prior to challenge. Furthermore, sera from mice immunized against the β-(Man)_3_-Fba-TT conjugate transferred protection against disseminated candidiasis to naïve mice, whereas *C. albicans*-preabsorbed immune sera did not, which confirmed that induced antibodies were protective. In conclusion, our results demonstrated that the addition of the TT to the vaccine conjugate provided sufficient self-adjuvanting activity without the need for additional adjuvant. This is the first report of which we are aware of a self-adjuvanting glycopeptide vaccine against any infectious disease. Prior to our work, immunostimulatory lipids conjugated to peptide antigens resulted in self-adjuvanting (lipopeptide) vaccines now tested in clinical trials [31], [32]. The lipid parts of the lipopeptide vaccines ranged from simple single fatty acid chains to complex lipids and glycolipids. Several self-adjuvanting mRNA vaccines, administered by an intradermal route, also induced anti-tumor immune responses [33]. We show in our study, however, that the coupling of a carrier protein component (TT) to the glycopeptide vaccine resulted in a self-adjuvanting conjugate, which lays the foundation for the novel concept of self-adjuvanting glycopeptide vaccines.

Protein carriers can influence the specificity, function, avidity, and idiotype of the antibody response to polysaccharide-protein conjugate vaccines [34]. We compared antibody isotype distribution of responses to both β-(Man)_3_ and Fba peptide epitopes in immune sera from mice vaccinated against β-(Man)_3_-Fba or β-(Man)_3_-Fba-TT with different adjuvant systems. IgM specific for both fungal epitopes was detected in sera of all immunized animals. IgG1 against the two epitopes was produced in sera of mice immunized with either β-(Man)_3_-Fba pulsed DC or β-(Man)_3_-Fba-TT with or without adjuvant. Interestingly, conjugation of TT to β-(Man)_3_-Fba led to the appearance of IgG2a specific for β-(Man)_3_ and Fba peptide epitopes. In contrast, the antibody responses to β-(Man)_3_-Fba without adjuvant, or with either alum or MPL as adjuvant consisted only of IgM antibodies. IgG1 and IgG2a were the major IgG subclasses against both the fungal glycan part and peptide parts of the β-(Man)_3_-Fba-TT conjugate, but when MPL was used as adjuvant, the IgG2a and IgG2b subclasses were detected with specificity for the β-(Man)_3_ epitope only. In the mouse, IgG2a and IgG3 efficiently fix complement and promote opsonophagocytosis [35]–[38], and IgG2a binds to the high-affinity macrophage Fcγ receptor [39]. These considerations may well explain the higher level of protection against candidiasis that was observed in mice immunized against the β-(Man)_3_-Fba-TT with or without adjuvant as compared to those that received the β-(Man)_3_-Fba formulation with adjuvant. Although the mechanism of protection afforded by antibodies specific for the Fba peptide part of the vaccine is under investigation, the importance of complement fixation for protection provided by antibodies specific for the β-(Man)_3_ has been established [18], [19]. These results show that TT not only enhances the immunogenicity of the glycopeptide vaccine, but also obviates the use of adjuvants.

Antibodies induced by non-DC/CFA approaches are apparently responsible for the protection against disseminated candidiasis. This was demonstrated by passive transfer of immune serum, which conferred protection to naive animals. Moreover, such protection was not conferred by normal serum from non-immunized mice or by immune serum pre-absorbed by *C. albicans* yeast forms. These results were not surprising since our previous work showed that immune serum from mice immunized against the fungal glycan [25], [29], monoclonal antibodies specific for the glycan [25], [29], [40], and monoclonal antibody specific for the Fba peptide component [17] of the vaccine all conferred protection when given to naïve animals.

The efficacy of the β-(Man)_3_-Fba-TT conjugate vaccine in prolonging the survival of mice after a lethal challenge with *C. albicans* was also demonstrated in outbred mice. The β-(Man)_3_-Fba-TT conjugate vaccine was immunogenic in Swiss Webster mice in the absence of an adjuvant, eliciting strong glycan- and peptide-specific antibodies and induced protection against candidiasis. Vaccine-mediated protection in this outbred mouse model was associated with a reduction in the levels of CFU in kidneys. Taken together, we found no evidence that protection had an MHC bias as evidenced by vaccine efficacy in BALB/c and C57BL/6 mice, and the vaccine effectiveness in outbred mice provides further support that this formulation may induce protection in humans as well. We also expect that establishment of active immunity when the host is immunologically normal will then protect that host upon an immunocompromised event later. This expectation is based on our findings that antibodies specific for the glycan part of the β-(Man)_3_-Fba-TT vaccine enhanced resistance to disseminated candidiasis of normal and neutropenic mice [29], [41]. This is an important fundamental question because a prevalent risk factor for enhancement of susceptibility to disseminated candidiasis in humans and mice is neutropenia.

Detection of specific antibodies induced by the β-(Man)_3_-Fba-TT vaccine were determined by ELISA in which wells of the plate were coated with either β-(Man)_3_ conjugated to bovine serum albumin or Fba peptide as a MAP conjugate to detect anti-glycan and peptide antibodies, respectively. We also confirmed the specificity of the response to both the glycan and peptide epitopes by inhibition ELISA, in which free soluble β-(Man)_3_ or Fba peptide inhibited the binding of antibodies in immune sera in a dose-dependent manner (data not shown). The binding of the specific antibodies to the actual fungal cell surface was confirmed by flow cytometric analyses, which demonstrated binding to *C. albicans* yeast forms, and by indirect immunofluorescence microscopy showing antibody reactivity with yeast and hyphal forms, which is consistent with our reported observations on monoclonal antibodies specific for the two fungal epitopes [17], [25].

In other work, conjugates made of the synthetic glycan covalently linked to tetanus toxoid was immunogenic in rabbits [42], [43], but was poorly immunogenic in mice [43]. In the present work, the conjugate became highly immunogenic upon inclusion of the Fba peptide [β-(Man)_3_-Fba-TT]. Since the Fba peptide alone induces protection [7], [17], its' inclusion in the vaccine construct not only increases immunogenicity, but also provides the host with a dual immune recognition formulation to ensure protection even against *C. albicans* mutants that may have lost the ability to express one of the two vaccine epitopes. Additional rationale for keeping the glycan as part of the vaccine is that, although the Fba peptide is unique to *C. albicans*, responses against the β-(Man)_3_ would be expected to protect against infection with a variety of other clinically important *Candida* species [15], including *C. tropicalis* [29], [44], *C. lusitaniae* [45], *C. guilliermondii* [46], [47] and the majority of *C. glabrata* strains [48], [49].

In summary, the development of an effective vaccine against disseminated candidiasis represents an alternative to the often ineffective antifungal drug therapeutic approach to management of this disease. The findings in the present study indicate that the β-(Man)_3_-Fba-TT glycopeptide vaccine is a promising candidate for disease prevention. In a recent study, we obtained evidence that antibody responses to at least the Fba component of the vaccine can be expected to occur even in mice modified to possess gastrointestinal tract colonization with the fungus and are producing serum antibodies in response to the colonization [16]. This modified mouse model more closely simulates the situation in most humans, thus providing additional confidence for going forward with our vaccine approach in humans.

## Materials and Methods

### *Candida albicans* strains

*C. albicans* 3153A and SC5314 were grown as stationary-phase yeast cells in glucose-yeast extract-peptone broth at 37°C, washed and suspended to the appropriate cell concentration (5×10^6^/ml) in Dulbecco's PBS (DPBS; Sigma), and used to infect mice intravenously (i.v.) as described [29], [40]. *C. albicans* strain 3153A was used also for serum antibody absorption, immunofluorescence staining and flow cytometric analysis.

### Mouse strains

Inbred strains BALB/c and C57BL/6, and outbred Swiss Webster (ND4) female mice (NCI Animal Production Program or Harlan) 5 to 7 weeks old were used. Mice were maintained and handled in accordance with a protocol (#150) approved by the Institutional Animal Care & Use committee (IACUC) regulations at Children's Hospital Research Institute in New Orleans.

### Synthesis of the Conjugate Vaccine

The glycoconjugate vaccine (**GV**) and the control vaccine lacking the mannotriose component (**CV**) were synthesized from the advanced building blocks **1**–**3** (Figure 8). The β-1,2 mannotriose derivatized with a triethylene glycol spacer **1** was synthesized as previously described [50]. The T-cell tetradecameric peptide (Fba) was assembled on a peptide synthesizer and a triethylene glycol tether was introduced at the C terminal end followed by a single lysine residue which was derivatized on its side chain by a thioacetic acid residue. This gave the building blocks **2a** or **2b**. Bromoacetate groups were introduced on approximately 20 of the lysine residues present in tetanus toxoid (State Serum Institute, Cophenhagen) to give **3**. **GV** was assembled by reacting **1** with **2a** and then conjugating this product with **3**. **CV** was prepared by conjugating 2b with 3. A detailed account of this synthesis will be reported elsewhere (Cartmell *et al.* Carbohydr. Res submitted).

### Immunizations of mice

Conjugates β-(Man)_3_-Fba or β-(Man)_3_-Fba-TT were administered at a subcutaneous (s.c.) location in the nape of the neck. The conjugates were given alone or as a mixture made either with alum (aluminum hydroxide gel, Sigma) or MPL (Lipid A, monophosphoryl, Sigma) or as a combination of both adjuvants. Negative control groups of mice were given DPBS buffer or adjuvant only. Immunization doses and schedules were 100 µl of 2.5 µg or 10 µg of either conjugate alone, or as a mixture containing either conjugate along with 50 µg alum or 10 µg MPL on days 1, 21 and 62. In some experiments the two adjuvants were combined and mixed with antigen for the priming and booster doses. Serum samples were collected 14 days after each immunization and tested by ELISA.

### Serological assays

Immune sera were analyzed for antibody titers after each immunization as described below. Although the titers increased after each dosing, the most profound changes were usually observed after the first booster, which is the result we chose to show for comparison of vaccine responses between the various groups.

For DC/CFA-based immunizations, DCs were pulsed *in vitro* with β-(Man)_3_-Fba vaccine as described [7]. The mice were given a priming dose and boosted at day 14 with fresh antigen-pulsed DCs and boosted a second time at day 28 with antigen (2.5 µg) emulsified in complete Freund adjuvant (CFA) given subcutaneously (s.c.). Control groups consisted of mice given DPBS, DC, or DC+CFA alone at the time of priming and boosters. For β-(Man)_3_-Fba, Fba-TT or (β-(Man)_3_-Fba-TTadministered alone or with alum or MPL, control groups were given adjuvant alone or DPBS buffer. Serum samples were collected 14 days after each immunization, diluted 1∶100 and tested by ELISA on plates coated with cell wall mannan extract from *C. albicans*, or Fba-MAP peptide (GenScript) or β-(Man)_3_-BSA as previously described [7], [16]. Briefly, *C. albicans* mannan extract [51], which is composed mainly of mannan, or synthetic β-(Man)_3_ coupled to BSA was dissolved at 4 µg/ml in carbonate buffer (pH 9.6); Fba-MAP (GenScript) was dissolved at 10 µg/ml in carbonate coating buffer (pH 9.5). Each was used to coat 96-well ELISA plates for testing duplicate 1∶100 dilutions of samples of each immune serum and control sera. Color development for each well was achieved by secondary antibody, goat anti-mouse polyvalent immunoglobulin (IgG, IgA, IgM) peroxidase conjugated antibody (diluted 1∶10,000 in PBST) (Sigma) and substrate (*O*-phenylenediamine and H_2_O_2_); OD reading was determined at 492 nm.

To determine dilution endpoint ELISA titers, serial 2-fold dilutions of sera in blocking buffer were prepared. The endpoint ELISA titer was taken as the reciprocal of the last serum dilution with an OD reading at least two-fold greater than the mean OD of negative control samples plus twice the standard deviation.

For antibody isotype and subclass determinations, peroxidase-conjugated rabbit anti-mouse heavy chain specific IgM, IgG1, IgG2a, IgG2b, and IgG3 (Rockland, PA) were diluted 1∶10,000 in blocking buffer and added to the appropriate wells, followed by addition of *O*-phenylenediamine substrate and H_2_O_2_ for color development and absorbance as before.

### Fungal challenge and assessment of protection

Two weeks after the second boost, immune and control mice were infected i.v. with a lethal dose of live *C. albicans* yeast cells (5×10^5^ in 0.1 ml of DPBS) prepared as described above and as before [7]. Passively immunized mice (below) also received the same challenge dose. Protection was evaluated by monitoring animal survival for 50–120 days, depending on the experiment. The mice were monitored for development of a moribund state, defined as being listless, disinterested in food or water, and nonreactive to finger probing. At the time that a mouse was deemed moribund, it was sacrificed and their kidneys were homogenized in DPBS and plated onto a nutrient agar to determine colony forming units (CFUs). After 50–120 days, the experiments were terminated and all the survivors at that time were sacrificed and their kidneys were assessed for CFU as before. The lowest limit of detection for the CFU assay was 50 CFU per kidney pair.

### Passive transfer of immune sera

Immune sera were obtained from vaccinated mice, pooled and stored at −20°C or absorbed before freezing with *C. albicans* 3153A yeast cells as before [7], [17], [29]. Immune sera pre-absorbed with yeast or DBPS buffer were used as passive transfer negative controls. Pre-absorbed immune sera were tested and found negative for antibodies against both β-(Man)_3_ and Fba peptide by ELISA as described above. Naïve BALB/c mice received 0.5 ml at an intraperitoneal (i.p.) location of full-strength immune serum or control serum or DBPS buffer. Four hours later, all mice were challenged i.v. with *C. albicans* (5×10^5^ yeast cells). All animals received a second dose (200 µl) of serum or negative control material i.p. 24 h after the first dose. Infected mice were sacrificed when they became moribund and their kidneys were assessed for CFUs as above.

### Flow cytometric analysis and immunofluorescence microscopy

Distribution of the β-(Man)_3_ and Fba peptide epitopes on yeast cells was determined by use of immune serum for flow cytometric analysis and indirect immunofluorescence. One hundred microliters of immune serum (1∶100 dilution in 1% BSA/DPBS) was added to a pellet of *C. albicans* yeast cells (5×10^6^) that was prewashed with DPBS buffer three times. The yeast cells were suspended in the immune serum [from β-(Man)_3_-Fba-TT immunized mice] preparation and incubated while shaking by rotation at room temperature (RT, 22–24°C) for 1–2 h. After incubation, the yeast cells were washed with DPBS three times, suspended in 200 µl of fluorescein- labeled goat anti-mouse IgM (u-chain specific; Sigma) (stock solution, 1 mg/ml; working solution, 20 µg/ml of DPBS) and incubated at RT described above for 0.5 h. The yeast cells were washed with DPBS three times and suspended in 500 µl of DPBS. Flow cytometry was performed using a BD Biosciences FACSVantage SE equipped with an argon laser excitation at 488 nm. 10,000 cells in each sample were analyzed (CellQuest Pro software)

For immunofluorescence assays, the fungal cells were immunostained and washed as described above and suspended in the 200 µl DPBS buffer. The cells were observed by confocal microscopy (LSM 510, Zeiss). The distribution of the β-(Man)_3_ and Fba peptide epitopes on the yeast cell surface was compared to that obtained with yeast cells fluorescently stained for detection of the MAb B6.1 epitope. Negative controls included showing non-reactivity of an irrelevant isotype control IgM MAb S-9 [52] (data not shown) and use of fluorescein-labeled goat anti-mouse secondary antibody only. As an additional control, pre-absorbed immune serum prepared as described above was tested for the binding reactivity to the *C. albicans* cell surface.

### Statistical analysis

Data were analyzed by GraphPad Prism 4 software (GraphPad Inc.). ELISA data were assessed for statistical significance by curve fit analysis. Differences in median survival time and in survival rates in *C. albicans*–challenged mice were analyzed by nonparametric two-tailed Mann-Whitney U test or Fisher's exact test, respectively. Differences in survival curves were assessed by the log-rank test. Data from CFU counts, in both in vitro and in vivo experiments, were analyzed by two-tailed Student's *t* test. Multiple comparisons were made by analysis of variance (one-way ANOVA) followed by Newman-Keuls post-test.

## Supporting Information

Figure S1 **β-(Man)_3_-Fba pulsed DC/CFA vaccine induced protective responses in C57BL/6 mice against disseminated candidiasis.** (A) Vaccination with β-(Man)_3_-Fba by the DC/CFA-based approach induced protection against disseminated candidiasis by *C. albicans* strain 3153A in C57BL/6 mice. Vaccinated mice had a prolonged survival time as compared to control mice that received DCs+CFA, DCs alone or DPBS (*P*<0.001). (B) Immunized mice also had greatly reduced or non-detectable CFU in their kidneys as compared to control mice (*P*<0.01). (C) Pooled serum from immune mice transferred protection to naïve mice. Note that the immunized mice had a similar survival curve as the naïve mice that received the immune serum. (D) Immunized mice and mice that received antiserum had significantly fewer or non-detectable CFU in kidneys as compared to the control groups that received either the immune serum that was preabsorbed with *C. albicans* yeast cells, or DPBS buffer (*P*<0.001). (E) Vaccination induced significant protection regardless of the fungal strain. Similar protection patterns were obtained when immunized mice were challenged with *C. albicans* strains SC5314 and 3153A.
(TIF)

## References

[1] Hsu FC, Lin PC, Chi CY, Ho MW, Wang JH (2009). Prognostic factors for patients with culture-positive Candida infection undergoing abdominal surgery.. J Microbiol Immunol Infect.

[2] Horn DL, Neofytos D, Anaissie EJ, Fishman JA, Steinbach WJ (2009). Epidemiology and outcomes of candidemia in 2019 patients: data from the prospective antifungal therapy alliance registry.. Clin Infect Dis.

[3] Zaoutis TE, Argon J, Chu J, Berlin JA, Walsh TJ (2005). The epdiemiology and attributable outcomes of candidemia in adults and children hospitalized in the United States: A propensity analysis.. Clin Infect Dis.

[4] Mochon AB, Cutler JE (2005). Is a vaccine needed against *Candida albicans*?. Med Mycol.

[5] Cassone A, De Bernardis F, Torosantucci A (2005). An outline of the role of anti-Candida antibodies within the context of passive immunization and protection from candidiasis.. Curr Mol Med.

[6] Cutler JE, Deepe GS, Klein BS (2007). Advances in combating fungal diseases: Vaccines on the threshold.. Nat Rev Microbiol.

[7] Xin H, Dziadek S, Bundle DR, Cutler JE (2008). Synthetic glycopeptide vaccines combining β-mannan and peptide epitopes induce protection against candidiasis.. Proc Natl Acad Sci USA.

[8] Cutler JE (2005). Defining criteria for anti-mannan antibodies to protect against candidiasis.. Curr Mol Med.

[9] De Bernardis F, Boccanera M, Adriani D, Spreghini E, Santoni G (1997). Protective role of antimannan and anti-aspartyl proteinase antibodies in an experimental model of *Candida albicans* vaginitis in rats.. Infect Immun.

[10] Matthews RC, Rigg G, Hodgetts S, Carter T, Chapman C (2003). Preclinical assessment of the efficacy of mycograb, a human recombinant antibody against fungal HSP90.. Antimicrob Agents Chemother.

[11] Yang Q, Wang L, Lu DN, Gao RJ, Song JN (2005). Prophylactic vaccination with phage-displayed epitope of *C. albicans* elicits protective immune responses against systemic candidiasis in C57Bl/6 mice.. Vaccine.

[12] Cassone A, Rappuol R (2010). Universal vaccines: shifting to one for many.. mBio.

[13] Bromuro C, Romano M, Chiani P, Berti F, Tontini M (2010). Beta-glucan-CRM 197 conjugates as candidates antifungal vaccines.. Vaccine.

[14] Krcmery V, Barnes AJ (2002). Non-albicans Candida spp. causing fungaemia: pathogenicity and antifungal resistance.. J Hosp Infect.

[15] Perlroth J, Cho B, Spellberg B (2007). Nosocomial fungal infections: epidemiology, diagnosis, and treatment.. Med Mycol.

[16] Cutler JE, Corti M, Lambert P, Ferris M, Xin H (2011). Horizontal transmission of *Candida albicans* and evidence of a vaccine response in mice colonized with the fungus.. PLoS One.

[17] Xin H, Cutler JE (2011). Vaccine and Monoclonal Antibody That Enhance Mouse Resistance to Candidiasis.. Clin Vaccine Immunol.

[18] Han Y, Kozel TR, Zhang MX, MacGill RS, Carroll MC (2001). Complement is essential for protection by an IgM and an IgG3 monoclonal antibody against experimental hematogenously disseminated candidiasis.. J Immunol.

[19] Xin H, Cutler JE (2006). Hybricoma passage in vitro may result in reduced ability of antimannan antibody to protect against disseminated candidiasis.. Infect Immun.

[20] Kuroda E, Sugiura T, Zeki K, Yoshida Y, Yamashita U (2000). Sensitivity difference to the suppressive effect of prostaglandin E_2_ among mouse strains: a possible mechanism to polarize Th2 type response in BALB/c mice.. J Immunol.

[21] Ashman RB (1990). Murine candidiasis: Susceptibility is associated with the induction of T cell-mediated, strain-specific autoreactivity.. Immunol Cell Biol.

[22] Ashman RB (1997). Genetic determination of susceptibility and resistance in the pathogenesis of *Candida albicans* infection.. FEMS Microbiology Letters.

[23] Hector RF, Domer JE, Carrow EW (1982). Immune responses to *Candida albicans* in genetically distinct mice.. Infect Immun.

[24] Didierlaurent A, Morel S, Lockman L, Giannini SL, Bisteau M (2009). AS04, an aluminum salt- and TLR4 agonist-based adjuvant system, induces a transient localized innate immune response leading to enhanced adaptive immunity.. J Immunol.

[25] Han Y, Kanbe T, Cherniak R, Cutler JE (1997). Biochemical characterization of *Candida albicans* epitopes that can elicit protective and nonprotective antibodies.. Infect Immun.

[26] Nitz M, Ling C-C, Otter A, Cutler JE, Bundle DR (2002). The unique solution structure and immunochemistry of the *Candida albicans* β-1,2-mannopyranan cell wall antiegns.. J Biol Chem.

[27] Casadevall A, Pirofski L-A (2007). Antibody-mediated protection through cross-reactivity introduces a fungal heresy into immunological dogma.. Infect Immun.

[28] Casadevall A, Dadachova E, Pirofski L-A (2004). Passive antibody therapy for infectious diseases.. Nature Reviews.

[29] Han Y, Cutler JE (1995). Antibody response that protects against disseminated candidiasis.. Infect Immun.

[30] Han Y, Morrison RP, Cutler JE (1998). A vaccine and monoclonal antibodies that enhance mouse resistance to *Candida albicans* vaginal infection.. Infect Immun.

[31] Moyle PM, Toth I (2008). Self-adjuvanting lipopeptide vaccines.. Curr Med Chem.

[32] Brown LE, Jackson DC (2005). Lipid-based Self-Adjuvanting Vaccines.. Current Drug Delivery.

[33] Fotin-Mleczek M, Duchardt KM, Lorenz C, Pfeiffer R, Ojkic-Zrna S (2011). Messenger RNA-based vaccines with dual activity induce balanced TLR-7 dependent adaptive immune responses and provide antitumor activity.. J Immunother.

[34] Maittra RW, Datta K, Lees A, Belouski SS, Pirofski L-A (2004). Immunogenicity and efficacy of *Cryptococcus neoformans* capsular polysaccharide glucuronoxylomannan peptide mimotope-protein conjugates in human immunoglobulin transgenic mice.. Infect Immun.

[35] Germann T, Bongartz M, Dlugonska H, Hess H, Schmitt E (1995). Interleukin-12 profoundly up-regulates the synthesis of antigen-specific complement-fixing IgG2a, IgG2b and IgG3 antibody subclasses *in vivo*.. Eur J Immunol.

[36] Nussbaum G, Yuan R, Casadevall A, Scharff MD (1996). Immunoglobulin G3 blocking antibodies to the fungal pathogen *Cryptococcus neoformans*.. J Exp Med.

[37] Schreiber JR, Cooper LJN, Diehn S, Dahlhauser PA, Tosi MF (1993). Variable region-identical monoclonal antibodies of different IgG subclass directed to *Pseudomonas aeruginosa* lipopolysaccharide O-specific side chain fuction differently.. JID.

[38] Cooper LJN, Schimenti JC, Glass DD, Greenspan NS (1991). H chain C domains influence the strength of binding of IgG for streptococcal group A carbohydrate.. J Immunol.

[39] Unkeless JC, Sciqliano E, Freedman VH (1988). Structure and function of human and murine receptors for IgG.. Annu Rev Immunol.

[40] Han Y, Riesselman MH, Cutler JE (2000). Protection against candidiasis by an immunoglobulin G3 (IgG3) monoclonal antibody specific for the same mannotriose as an IgM protective antibody.. Infect Immun.

[41] Han Y, Cutler JE (1997). Assessment of a mouse model of neutropenia and the effect of an anti-candidiasis monoclonal antibody in these animals.. J Infect Dis.

[42] Wu X, Bundle DR (2005). Synthesis of glycoconjugate vaccines for *Candida albicans* using novel linker methodology.. J Org Chem.

[43] Wu X, Lipinski T, Carrel FR, Bailey JJ, Bundle DR (2007). Synthesis and immunochemical studies on a *Candida albicans* cluster glycoconjugate vaccine.. Org Biomol Chem.

[44] Kobayashi H, Matsuda K, Ikeda T, Suzuki M, Takahashi S (1994). Structures of cell wall mannans of pathogenic *Candida tropicalis* IFO 0199 and IFO 1647 yeast strains.. Infect Immun.

[45] Shibata N, Kobayashi H, Okawa Y, Suzuki S (2003). Existence of novel β-1,2 linkage-containing side chain in the mannan of *Candida lusitaniae*, antigenically related to *Candida albicans*.. Eur J Biochem.

[46] Shibata N, Akagi R, Hosoya T, Kawahara K, Suzuki A (1996). Existence of novel branched side chains containing β-1,2 and α-1,6 linkages corresponding to antigenic factor 9 in the mannan of *Candida guilliermondii*.. Journal of Biological Chemistry.

[47] Suzuki A, Shibata N, Suzuki M, Saitoh F, Oyamada H (1997). Characterization of β-1,2-mannosyltransferase in *Candida guilliermondii* and its utilization in the synthesis of novel oligosaccharides.. J Biol Chem.

[48] Goins T, Cutler JE (2000). Relative abundance of oligosaccharides in *Candida* species as determined by fluorophore-assisted carbohydrate electrophoresis.. J Clin Microbiol.

[49] Kobayashi H, Mitobe H, Takahashi K, Yamamoto T, Shibata N (1992). Structural study of a cell wall mannan-protein complex of the pathogenic yeast *Candida glabrata* IFO 0622 strain.. Arch Biochem Biophys.

[50] Dziadek S, Jacques S, Bundle DR (2008). A novel linker methodology for the synthesis of tailored conjugate vaccines composed of complex carbohydrate antigens and specific T_H_-cell peptide epitopes.. Chemistry.

[51] Kanbe T, Cutler JE (1994). Evidence for adhesin activity in the acid-stable moiety of the phosphomannoprotein cell wall complex of *Candida albicans*.. Infect Immun.

[52] Pincus SH, Shigeoka AO, Moe AA, Ewing LP, Hill HR (1988). Protective efficacy of IgM monoclonal antibodies in experimental group B streptococcal infection is a function of antibody.. J Immunol.

