# Supplementary figures and images for: Self-Adjuvanting Glycopeptide Conjugate Vaccine against Disseminated Candidiasis
Source: PLoS One. 2012 Apr 26;7(4):e35106. doi: 10.1371/journal.pone.0035106 (PMC3338514; doi:10.1371/journal.pone.0035106)

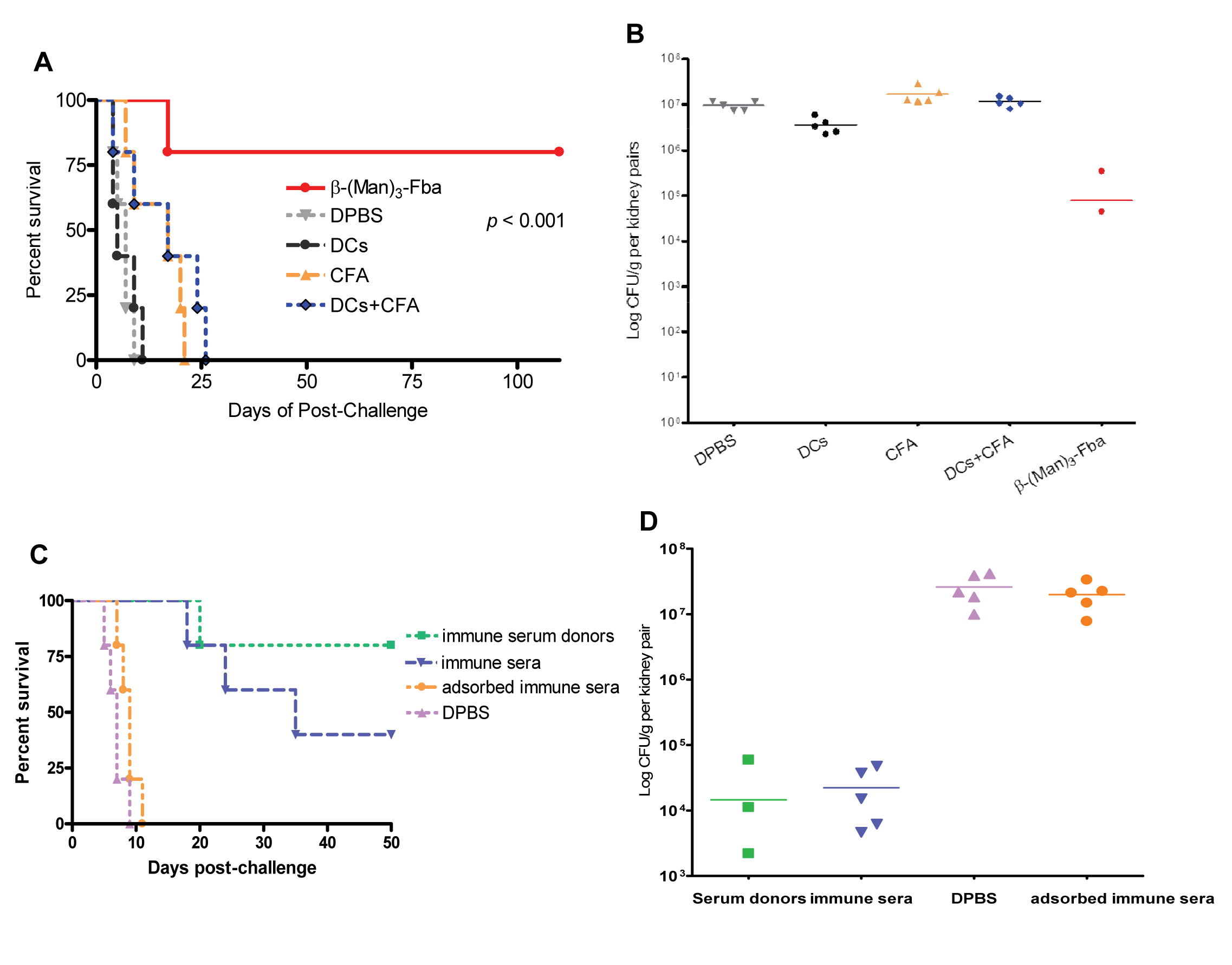

Supplement: Figure S1 — β-(Man)3-Fba pulsed DC/CFA vaccine induced protective responses in C57BL/6 mice against disseminated candidiasis. (A) Vaccination with β-(Man)3-Fba by the DC/CFA-based approach induced protection against disseminated candidiasis by C. albicans strain 3153A in C57BL/6 mice. Vaccinated mice had a prolonged survival time as compared to control mice that received DCs+CFA, DCs alone or DPBS (P<0.001). (B) Immunized mice also had greatly reduced or non-detectable CFU in their kidneys as compared to control mice (P<0.01). (C) Pooled serum from immune mice transferred protection to naïve mice. Note that the immunized mice had a similar survival curve as the naïve mice that received the immune serum. (D) Immunized mice and mice that received antiserum had significantly fewer or non-detectable CFU in kidneys as compared to the control groups that received either the immune serum that was preabsorbed with C. albicans yeast cells, or DPBS buffer (P<0.001). (E) Vaccination induced significant protection regardless of the fungal strain. Similar protection patterns were obtained when immunized mice were challenged with C. albicans strains SC5314 and 3153A. (TIF) [file pone.0035106.s001.tif]
